# Adaptive Antithrombotic Strategy for Tandem Occlusion Stroke: Escalating Therapy After Thrombectomy and Stenting

**DOI:** 10.3390/diagnostics16091281

**Published:** 2026-04-24

**Authors:** Gregory Howaldt, Mara Thut, Miklos Krepuska, Patrick Thurner, Jawid Madjidyar, Anna Kyselyova, Susanne Wegener, Christoph Globas, Andreas Luft, Tilman Schubert, Lars Michels, Zsolt Kulcsar

**Affiliations:** 1 Faculty of Medicine, University of Zurich, 8006 Zurich, Switzerland; mara.thut@usz.ch; 2 Department of Neuroradiology, Clinical Neurocenter, University Hospital Zurich, Frauenklinikstrasse 10, 8091 Zurich, Switzerland; miklos.krepuska@usz.ch (M.K.); patrick.thurner@usz.ch (P.T.); jawid.madjidyar@usz.ch (J.M.); anna.kyselyova@usz.ch (A.K.); tilman.schubert@usz.ch (T.S.); lars.michels@usz.ch (L.M.); 3 Department of Neurology, Clinical Neurocenter, University Hospital Zurich, Frauenklinikstrasse 26, 8091 Zurich, Switzerland; susanne.wegener@usz.ch (S.W.); christoph.globas@usz.ch (C.G.); andreas.luft@usz.ch (A.L.); 4 Cerneo Center for Neurology and Rehabilitation, Seestrasse 18, 6354 Vitznau, Switzerland

**Keywords:** tandem occlusion, acute stroke, thrombectomy, carotid stenting, antithrombotic strategy

## Abstract

**Background/Objectives**: There is no consensus on standardized treatment algorithms for patients with acute ischemic stroke due to anterior circulation tandem occlusions. This study evaluated the outcomes of mechanical thrombectomy and carotid artery stenting in such patients, with a particular focus on a standardized, adaptive, and escalating antithrombotic strategy. **Methods**: This single-center retrospective study included patients with atherosclerotic tandem occlusion treated between January 2019 and July 2023 at our comprehensive stroke center. All patients underwent mechanical thrombectomy and carotid artery stenting and received a standardized antithrombotic regimen, including the administration of the GPIIb/IIIa antagonist eptifibatide as rescue therapy. **Results**: Sixty-seven patients were included in the analysis. Thirty-five patients (52.2%) received eptifibatide due to acute stent thrombosis. Subtotal to total revascularization (mTICI 2b-3) was achieved in 98.5% of patients. The carotid artery reocclusion rate was 3.4% at discharge. Symptomatic intracranial hemorrhage occurred more frequently in patients treated with eptifibatide (9.0% vs. 0%, *p* = 0.021) but was not associated with mortality or favorable outcome (mRS 0–2) at 90 days. In univariable regression analysis, eptifibatide administration was not significantly associated with symptomatic intracranial hemorrhage (OR 1.9, 95% CI 0.3–11.4; *p* = 0.465). Older age was associated with mortality. **Conclusions**: Our adaptive antithrombotic protocol demonstrated high revascularization and low carotid reocclusion rates. Rescue use of eptifibatide was not significantly associated with symptomatic intracranial hemorrhage; however, a clinically relevant risk cannot be excluded. These findings highlight the importance of tailored antithrombotic strategies in acute ischemic stroke to maintain stent patency while minimizing hemorrhagic complications.

## 1. Introduction

Approximately 10–15% of acute ischemic strokes due to large vessel occlusion (LVO) are caused by tandem occlusions (TOs), i.e., associated with high-grade stenosis or occlusion of the ipsilateral cervical internal carotid artery (ICA) [1]. Left untreated, TOs are associated with poor clinical outcomes, including high rates of disability and mortality [2].

Despite well-defined standards for mechanical revascularization in acute LVO-related stroke [3], the optimal treatment strategy for tandem occlusions remains uncertain. There are several approaches for endovascular treatment, such as emergent carotid artery stenting (CAS) in conjunction with mechanical thrombectomy (MT), MT alone, or angioplasty without stenting, treating the intracranial LVO first and the carotid occlusion second, or vice versa [4]. However, there are very few studies investigating the outcome of these treatment options [5]. In addition to procedural considerations, the optimal antithrombotic strategy remains unclear, with no established standard.

The use of double antiplatelet treatment in atherosclerotic carotid artery disease (ACAD) treated by endovascular stent reconstruction is well-established: it prevents early occlusion, thromboembolic events and decreases restenosis of the cervical ICA lesion after stenting [6,7]. However, in the setting of acute ischemic stroke, the potential addition of thrombolytic therapy, the volume of the unsalvageable ischemic brain damage, as well as the possible complications of the endovascular therapy render treatment more complex. Nevertheless, antithrombotic agents carry a burden of risk for postinterventional hemorrhagic complications, and it remains challenging to balance those potential risks and advantages. Studies investigating the efficacy and safety of periprocedural antiplatelet therapy during MT for tandem lesions are scarce. Hardly any treatment guidelines exist due to the lack of randomized trials, causing a state of uncertainty and high variability in practice among stroke experts [8,9].

Our goal was to evaluate our standardized, adaptive, escalating treatment algorithm for TO in terms of outcome, efficacy and safety, with a particular focus on the rescue administration of the GPIIb/IIIa antagonist eptifibatide.

## 2. Materials and Methods

### 2.1. Study Design and Patient Selection

Patients were selected from our prospective mechanical thrombectomy database at our comprehensive stroke center. Data were prospectively collected from hospital records and subsequently analyzed retrospectively.

All living patients provided written informed consent. Deceased patients or those with unavailable consent were included under additional ethical approval permitting the use of anonymized clinical and imaging data in accordance with Swiss regulations and the Declaration of Helsinki. The study was approved by the regional ethics committee (KEK Zurich; ID 2022-00422).

Only patients with acute ischemic stroke due to atherosclerotic tandem occlusion were included. TO was defined as severe stenosis (≥90% NASCET) or occlusion of the cervical ICA in combination with an intracranial LVO, such as the ICA, M1, and M2 segments of the middle cerebral artery. Patients with TO due to carotid artery dissection were excluded.

All patients underwent initial clinical assessment by a stroke neurologist and standardized imaging, including parenchymal imaging (CT or MRI) and vascular imaging (CTA or MRA). Eligible patients received intravenous tissue plasminogen activator (IV tPA) Alteplase (Actilyse, Boehringer Ingelheim, Basel, Switzerland) at a dose of 0.9 mg/kg body weight before undergoing endovascular therapy (EVT).

The decision to proceed with endovascular treatment was made by an interdisciplinary team in accordance with current international guidelines [10]. Patients were stratified based on the use of eptifibatide, which was administered as rescue therapy in cases of suspected early rethrombosis during the procedure.

### 2.2. Interventional Procedure

Endovascular treatment was performed under general anesthesia. Patients who did not receive intravenous thrombolysis were given an initial intravenous bolus of heparin (2000–3000 IU) after vascular access was established.

A distal-to-proximal approach was preferred, with intracranial thrombectomy performed prior to CAS whenever feasible. Balloon guide catheters (Flowgate 8F, Stryker, Irvine, CA, USA, or Bobby, Microvention, Aliso Viejo, CA, USA) were used routinely. Thrombectomy was performed using aspiration catheters (Catalyst 5, 6 or 7, Stryker, Irvine, CA, USA; Sofia Plus, Microvention, Aliso Viejo, CA, USA), stent retrievers (Solitaire, Medtronic, Galway, Ireland; Trevo, Stryker, Irvine, CA, USA), or a combination of both, at the operator’s discretion.

Following intracranial recanalization, CAS was performed in all cases using dedicated carotid stents. (Precise Pro RX, Cordis, Miami, FL, USA; Casper, Microvention, Aliso Viejo, CA, USA). Balloon angioplasty was performed before and after stent deployment as required., using Emerge and NC Emerge balloon angioplasty catheters (sizes 2–3 × 20 mm and 4–5.5 × 20 mm, respectively; Boston Scientific, Galway, Ireland). All patients received 300 mg of intravenous aspirin prior to stent placement (Aspégic, Opella Healthcare AG, Rotkreuz, Switzerland).

Control angiography was performed approximately 15 min after stenting. The GPIIb/IIIa antagonist eptifibatide (Integrilin, GalaxoSmithKline AG, Münchenbuchsee, Switzerland) was administered intravenously in cases of suspected early rethrombosis, defined as new filling defects, in-stent contrast stagnation, or reduced antegrade flow (mTICI 0–1). A weight-based bolus (180 µg/kg) was followed by a continuous infusion (2 µg/kg/min), with dose adjustment in patients with impaired renal function, according to the recommendations in percutaneous coronary interventions. The infusion was continued for 6–24 h.

After follow-up imaging at 12–24 h, dual antiplatelet therapy with aspirin (100 mg) and clopidogrel (75 mg) was initiated, depending on infarct size.

**Figure 1:**
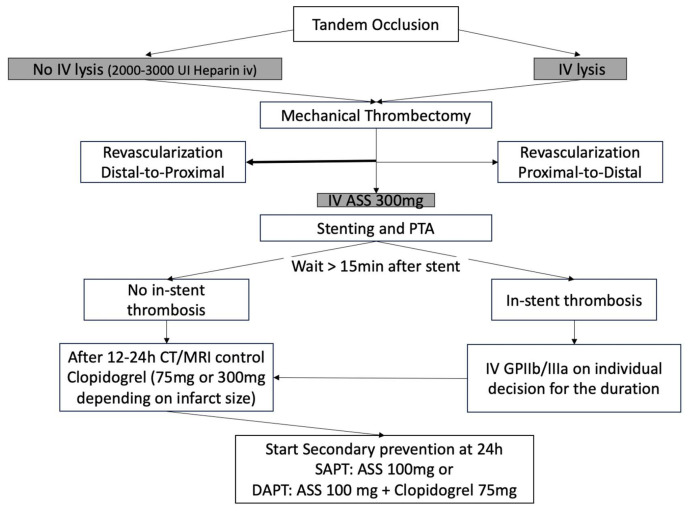
Antithrombotic medication regime of our institution in acute tandem occlusions. IV, intravenous; ASS, acetylsalicylic acid; DAPT, double oral antiplatelet therapy.

The institutional antithrombotic regimen for acute TO is illustrated in Figure 1.

### 2.3. Outcome Measures

At 90 days, the functional status was evaluated using the modified Rankin Scale (mRS), with a score of 0–2, indicating our primary and favorable outcome [11]. Based on the European Cooperative Acute Stroke Study II criteria, cerebral hemorrhages were categorized as either parenchymal hematoma (PH) or hemorrhagic infarction (HI) [12]. Any parenchymal hematoma, subarachnoid hemorrhage, or intraventricular hemorrhage linked to a 4-point or greater decline in the National Institutes of Health Stroke Scale (NIHSS) score was classified as symptomatic intracranial hemorrhage (sICH) [1]. The following secondary outcomes were observed: complete reperfusion (mTICI 3) and all-cause mortality at 90 days. Effective reperfusion was defined as a modified Thrombolysis In Cerebral Infarction (mTICI) score of 2b-3 [1]. Stent occlusion at hospital discharge and 90 days.

### 2.4. Statistical Analysis

Data were analyzed using SPSS (version 31; IBM Corp., Armonk, NY, USA). Continuous variables are presented as mean ± standard deviation or median (interquartile range), as appropriate, and were compared using the unpaired *t*-test or Mann–Whitney U test. Categorical variables are reported as counts (percentages) and were compared using Fisher’s exact test. For key proportions in Table 1, Table 2 and Table 3 (e.g., sICH, 90-day mortality, favorable outcome), we provide 95% confidence intervals based on exact (Clopper–Pearson) methods.

For the main analyses, available case data were used, and no multiple imputation was performed. The proportion of missing data was low for most baseline and procedural variables; patients with missing information on stent patency or 90-day outcomes were excluded from the respective analyses and are indicated in the tables.

Given the low number of symptomatic intracranial hemorrhage (sICH) events, we aimed to avoid model overfitting and therefore did not perform a fully adjusted multivariable analysis with multiple covariates. Instead, the crude association between periprocedural eptifibatide administration and sICH was first assessed using univariable logistic regression.

In additional exploratory sensitivity analyses, parsimonious logistic regression models were fitted, including eptifibatide and at most one clinically relevant covariate (age or baseline NIHSS), to account for potential confounding while respecting the limited number of events.

Odds ratios (ORs) with 95% confidence intervals (CIs) are reported, and a two-sided *p*-value < 0.05 was considered statistically significant. In accordance with the commonly recommended rule of at least 10 events per predictor, models were restricted to a maximum of two predictors (eptifibatide and one covariate).

Model fit was assessed descriptively using the −2 log-likelihood and, where applicable, the Hosmer–Lemeshow goodness-of-fit test.

## 3. Results

**Figure 2:**
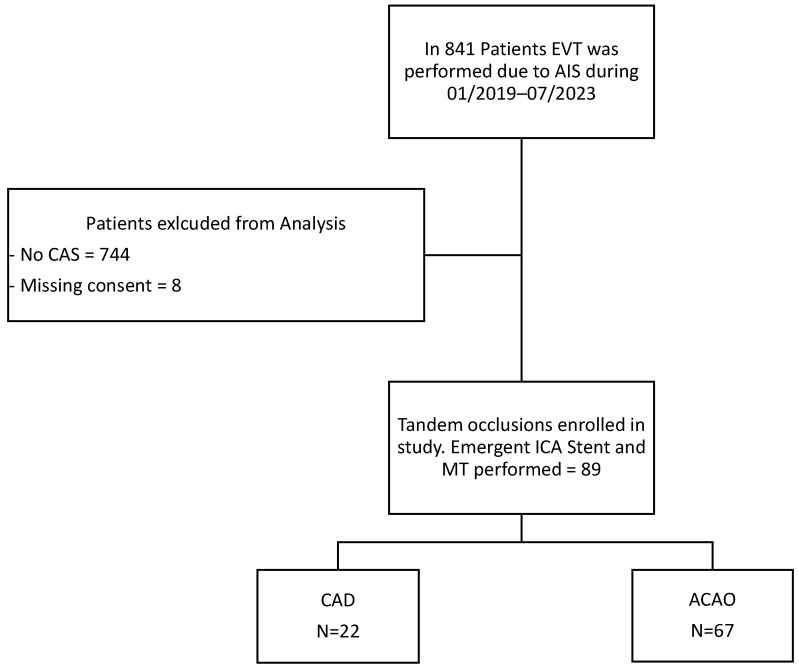
Flowchart of included patients for analysis. EVT, endovascular thrombectomy; AIS, acute ischemic stroke; ICA, internal carotid artery; CAD, carotid artery dissection; ACAO, atherosclerotic carotid artery occlusion.

Between January 2019 and July 2023, a total of 841 patients underwent endovascular treatment for acute ischemic stroke at our comprehensive stroke center. Among these, 92 patients presented with tandem lesions in the anterior circulation and were treated with MT. Patients who underwent isolated ICA stenting without thrombectomy were excluded. Eight additional cases were excluded due to missing consent. Of the remaining 89 patients (10.6%) with TO, 22 cases (24.7%) associated with carotid artery dissection were excluded. The final analysis included 67 patients (75.3%) with atherosclerotic TO (Figure 2).

Baseline characteristics are summarized in Table 1. The mean age of the study population was 74 years, and 70.1% of patients were male.

The median NIHSS score at admission was 11.5 (IQR 7–16). Intravenous thrombolysis was administered in 26 patients (38.8%). The median Alberta Stroke Program Early CT Score (ASPECTS) was 8 (IQR 7–9). Median onset-to-puncture time was 255 min (IQR 165–691), and median procedure time was 75 min (IQR 54–113).

### 3.1. Angiographic Outcomes

Successful reperfusion (mTICI 2b–3) was achieved in 66 of 67 patients (98.5%), while complete reperfusion (mTICI 3) was achieved in 27 patients (40.3%).

### 3.2. Periprocedural Antithrombotic Therapy

Intravenous aspirin (300 mg) was administered prior to CAS in all patients. Eptifibatide was used as rescue therapy in 35 patients (52.2%) due to suspected rethrombosis or in-stent thrombosis. The mean duration of eptifibatide administration was 13.6 h (range 0.5–26 h). Dual antiplatelet therapy was initiated within 24 h in 82.1% of patients.

### 3.3. Clinical Outcomes

At 90 days, a favorable outcome (mRS 0–2) was observed in 29 patients (43.2%). Twelve patients (17.9%) died during hospitalization, including six patients with symptomatic intracranial hemorrhage (sICH). An additional seven patients died within 90 days, resulting in an overall 90-day mortality rate of 28.4%.

**Figure 3:**
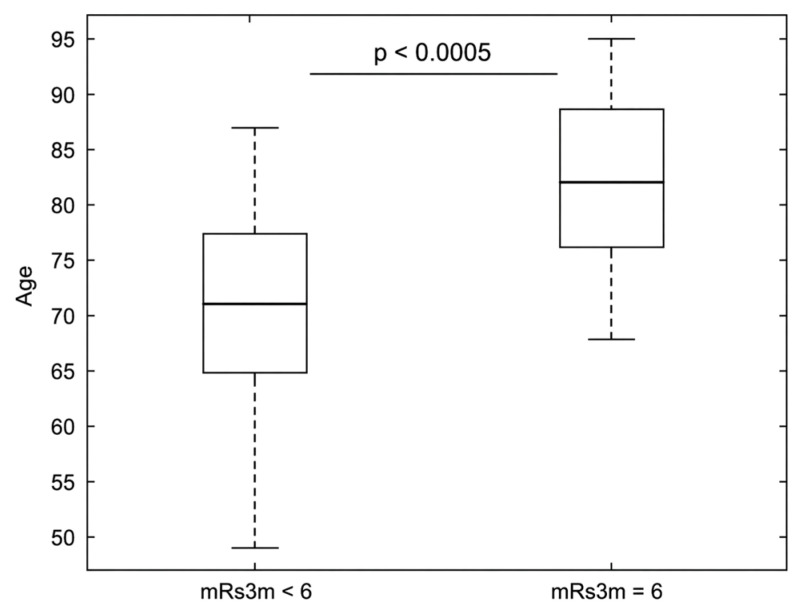
Boxplot Analysis comparing age distribution of surviving and deceased patients (mRS = 6) at 90 days (3m): Deceased Patients within 90 days were significantly older, showing a correlation between age and mortality. Loss of follow-up occurred in three patients; mRS, modified Rankin Scale score; 3m, 90 days.

Patients who died (mRS 6 at 90 days) were significantly older than survivors (Figure 3; mean age 82 vs. 70 *p* < 0.005).

Any intracranial hemorrhage on follow-up imaging occurred in 25 patients (37.7%). Symptomatic ICH occurred in 6 patients (9.0%), exclusively in the eptifibatide group (17.1% vs. 0%; *p* = 0.021). Among these patients, four had received intravenous thrombolysis with rtPA prior to the procedure, and five received intraprocedural heparin in addition to aspirin loading and eptifibatide. Individual patient characteristics are provided in Supplementary Table S1.

In univariable logistic regression, eptifibatide use was not significantly associated with sICH (OR 1.9, 95% CI 0.3–11.4; *p* = 0.465). In exploratory parsimonious models adjusted for age or baseline NIHSS, effect estimates remained numerically elevated but with wide confidence intervals crossing unity (age-adjusted OR 2.2, 95% CI 0.4–13.1; *p* = 0.395; NIHSS-adjusted OR 2.1, 95% CI 0.4–12.2; *p* = 0.429) (Table 4).

### 3.4. Stent Patency

Stent occlusion at hospital discharge was observed in 2 patients (3.4%). At 90 days, stent occlusion was observed in 4 patients, all of whom had received eptifibatide.

No significant difference was observed between single-layer and dual-layer stents regarding the need for rescue eptifibatide administration (16 vs. 18; *p* = 0.372).

### 3.5. Group Comparison

Comparative analysis between patients treated with and without eptifibatide showed no significant differences in baseline characteristics or clinical outcomes, including 90-day mortality and favorable outcome. However, all cases of sICH occurred in the eptifibatide group, resulting in a statistically significant difference between groups (6 vs. 0; *p* = 0.021) (Table 3).

## 4. Discussion

In this study of patients with atherosclerotic TO treated with combined CAS and MT, our adaptive escalating treatment protocol proved to be efficient in successful recanalization and prevention of early stent occlusions.

The protocol consisted of systematic periprocedural intravenous aspirin administration, with escalation to eptifibatide in cases of suspected stent thrombosis. Aspirin alone appeared sufficient to maintain stent patency in approximately half of the cases, with low rates of postprocedural hemorrhagic complications. Although unadjusted analyses suggested a higher incidence of sICH in patients receiving eptifibatide, this association was attenuated in regression analyses. Effect estimates remained numerically elevated but were accompanied by wide confidence intervals, indicating statistical uncertainty. Accordingly, these findings may point toward a potential increased risk but remain inconclusive and should be interpreted as exploratory.

The management of TO in acute ischemic stroke remains challenging due to the complexity of both intracranial and extracranial lesions. During intracranial revascularization, the presence of a severely diseased carotid artery increases the risk of plaque disruption and further embolization [13]. In addition, access to the intracranial occlusion may be technically demanding, particularly in cases requiring multiple thrombectomy attempts.

Once the procedure reaches the point of dealing with the carotid disease, new challenges are faced. In contrast to elective carotid interventions, patients are typically not pretreated with dual antiplatelet therapy, which increases the risk of early stent thrombosis and thromboembolic complications [14]. At the same time, intensified antithrombotic therapy in the acute stroke setting increases the risk of hemorrhagic transformation. Therefore, a careful balance between maintaining vessel patency and minimizing hemorrhagic risk is essential [15,16].

Our adaptive antithrombotic approach was designed to address this balance. In line with previous reports, our findings suggest that a minimalistic strategy using aspirin alone may be sufficient in the hyperacute phase for a subset of patients, potentially reducing hemorrhagic risk [17,18]. However, early angiographic signs of thrombosis after stent implantation may indicate a high risk of reocclusion and justify escalation of antithrombotic therapy [19,20].

We observed no significant difference in the frequency of eptifibatide use between single-layer and dual-layer carotid stents, although differences in thrombogenicity between stent types have been reported [21,22]. Overall, the protocol achieved a low rate of early stent reocclusion (3.4% at discharge), which is lower than rates reported in previous multicenter analyses. For example, Allard et al. reported occlusion rates of approximately 20% within 24–36 h [23]. Differences in antithrombotic regimens, including more frequent use of GPIIb/IIIa inhibitors in our cohort, may partly explain these findings.

Interestingly, higher rates of subtotal and total recanalization were observed in patients receiving GPIIb/IIIa inhibitors. This suggests that eptifibatide may contribute to maintaining vessel patency during the procedure. However, the observational design precludes causal interpretation of this association. Eptifibatide may play a role as a potent agent in preventing occlusion and ensuring recanalization. Moreover, preventing early stent occlusion appears to be an essential component of successful acute ischemic stroke treatment with CAS, as occlusions are associated with worse functional outcomes and increased mortality [23]. The median onset-to-puncture time in our cohort (255 min) is closely aligned with multicenter registry data on tandem occlusions, such as TITAN (246 min, IQR 185–320) and ETIS (260 min, IQR 180–331), indicating comparable workflow times despite the procedural complexity of these lesions [24].

Tandem lesions, like high-grade carotid artery stenosis or occlusion, carry the risk of revascularization-related hyper perfusion, aggravated by the size of the acute ischemic injury. This may play an essential role in the rate of reperfusion bleeding in our series. However, it is an important finding that sICH occurred exclusively in patients treated with eptifibatide, although no statistically significant association was observed in regression analyses. Our descriptive analysis of the six sICH cases suggests that combined exposure to intravenous thrombolysis, heparin, and eptifibatide might increase hemorrhagic risk, in line with prior work highlighting the bleeding risk of IVT plus heparin co-administration [25]. However, the small number of events precludes firm conclusions. The observed sICH rate (9.0%) is consistent with previously reported rates in similar populations, such as the TITAN registry analysis (7.3–9.8%) [26] or the meta-analysis of Sadeh-Gonik et al. (8%) [27]. Nevertheless, the clustering of hemorrhagic events in the eptifibatide group raises concerns regarding dosing strategies derived from coronary interventions, which may not be directly transferable to acute ischemic stroke. Dose adjustment strategies may therefore warrant further investigation.

The overall mortality rate in this cohort (28.4%) was relatively high compared with previous studies. One likely contributing factor is the advanced age of the study population (mean age of 74 years). Older age was significantly associated with mortality, underscoring its importance as a prognostic factor in this setting [28]. In comparison, prior registry data, including the pooled analysis of the TITAN and ETIS registries, reported younger patient populations (mean age, 62 years) and lower mortality rates (11.6%) [26], which may partly account for the observed differences. Similarly, previous studies on tandem occlusions have described mean patient ages of approximately 65 years among those treated with CAS-MT [11,24,26,29].

Despite increasing interest in TO, there is still no consensus regarding optimal revascularization strategies (angioplasty alone vs. CAS) or periprocedural antiplatelet management. Previous studies report divergent findings. Antiplatelet administration in TO treated with EVT was associated with better angiographic outcomes and decreased mortality, without increasing hemorrhagic complications, according to Zhu et al. [26]. In a retrospective analysis of 217 patients pretreated with antiplatelet agents following MT, no increased risk of ICH was observed [30]. On the other hand, a post hoc analysis of the MR CLEAN trial revealed that prior antiplatelet medication was associated with a higher incidence of ICH [16].

The complexity of TO likely contributes to their underrepresentation in randomized controlled trials. Ongoing studies, such as the TITAN trial, aim to evaluate different treatment strategies and antithrombotic approaches [1,28] and are expected to provide important insights into optimal management. However, in the absence of standardized protocols, variability in clinical practice is likely to persist.

With the aim of minimizing hemorrhagic risk while maintaining recanalization and stent patency, an adaptive, escalation-based protocol may represent a pragmatic approach in the management of TO. Given the relatively high rate of symptomatic bleeding observed in patients receiving GPIIb/IIIa inhibitors, both the choice of rescue agent (GPIIb/IIIa inhibitors vs. intravenous P2Y12 inhibitors) and dosing strategies—particularly those extrapolated from acute coronary interventions—warrant further evaluation.

Taken together, our findings support the concept of an adaptive antithrombotic strategy tailored to intraprocedural findings. While this approach may help balance the competing risks of thrombosis and hemorrhage, the safety and efficacy of specific agents and dosing regimens should be investigated in adequately powered prospective studies.

### Limitations

This study has several limitations. First, it was conducted at a single comprehensive stroke center with a specific workflow and antithrombotic protocol, which may limit the generalizability of the findings to other settings.

Second, the indication for eptifibatide was operator-dependent and based on intraprocedural findings, introducing potential selection and indication bias that cannot be fully adjusted for. Patients receiving rescue therapy likely had more complex vascular and procedural characteristics than those treated with aspirin alone, further limiting comparability between groups.

Third, the retrospective single-arm design without a randomized control group precludes causal inference regarding treatment effects. Accordingly, observed differences between groups should be interpreted with caution.

Fourth, the small number of sICH events (6/67) substantially limits statistical power. The resulting wide confidence intervals are compatible with both clinically relevant harm and no effect, indicating a considerable risk of type II error. Consequently, the analyses should be regarded as exploratory and hypothesis-generating. To mitigate overfitting, regression analyses were restricted to univariable and parsimonious models.

Finally, missing data were not imputed, which may have introduced bias if data were not missing at random, particularly for follow-up stent patency and 90-day favorable outcomes.

## 5. Conclusions

Our adaptive, escalating antithrombotic protocol was associated with high rates of revascularization and low rates of carotid reocclusion, supporting its potential effectiveness. Rescue treatment with intravenous GPIIb/IIIa antagonists was associated with numerically increased odds of sICH; however, wide confidence intervals indicate substantial uncertainty, and a clinically relevant effect cannot be excluded.

These findings support the use of GPIIb/IIIa antagonists as part of a tailored antiplatelet strategy during acute carotid stenting in tandem occlusions to maintain stent patency while balancing hemorrhagic risk. Further prospective studies are warranted to define optimal antiplatelet regimens in this setting.

## Figures and Tables

**Table 1:** Baseline characteristics of patients with acute ischemic stroke due to atherosclerotic tandem occlusion.

Variables	ACAO (*N* = 67)
Age mean (±SD)	74 (±15)
Male sex, no. (%)	47 (70.1)
Medical History	
Hypertension, no (%)	48 (71.6)
Hyperlipidemia, no. (%)	27 (40.3)
Diabetes mellitus, no. (%)	17 (25.4)
Atrial fibrillation, no. (%)	5 (7.5)
Smoking, no. (%)	30 (44.8)
Site of intracranial Occlusion *N* (%) *	
ICA terminus	2 (3.0)
MCA M1	39 (58.2)
MCA M2	15 (22.4)
MCA M3	9 (13.4)
ACA A1	2 (3.0)
ACA A2	1 (1.5)
Fetal PCA	1 (1.5)
Stroke characteristics and treatment	
NIHSS on admission, median (IQR)	11.5 (7–16)
ASPECTS, median (IQR)	8 (7–9)
IV thrombolysis, no. (%; 95% CI)	26 (38.8%; 27.1–51.5)
Onset to puncture min, median (IQR)	255.5 (165–691.5)
Procedure time min, median (IQR)	75 (54–113.5)
mTICI score 2b-3, no. (%; 95% CI)	66 (98.5%; 91.9–99.9)
mTICI 3, no. (%; 95% CI)	27 (40.3%; 28.6–53.1)
Number of stents, median (IQR)	1 (1–1)
Use of periprocedural antithrombotic agents *	
Eptifibatide, no. (%; 95% CI)	35 (52.2%; 39.7–64.5)
Eptifibatide use in hours, mean (min.–max.)	13.6 (0.5–26)
IVT rtPA, no. (%; 95% CI)	26 (38.8%; 27.1–51.5)
Heparin, no (%; 95% CI)	37 (55.2%; 42.8–67.6)
Clopidogrel loading min. 300 mg, no. (%; 95% CI)	25 (37.3%; 26.1–49.6)
Secondary prevention at 24 h	
DAPT: ASS + Clopidogrel, no. (%; 95% CI)	55 (82.1%; 70.0–90.6)

* Fourteen patients had combined treatment with rtPA and Eptifibatide. ACAO, atherosclerotic carotid artery occlusion; IVT rtPA, intravenous thrombolysis with recombinant tissue Plasminogen Activator; NIHSS, National Institutes of Health Stroke Scale; ASPECTS, Alberta Stroke Program Early CT Score; DAPT, Dual Antiplatelet Therapy. Data are presented as *n* (%). Proportions include exact 95% confidence intervals calculated using the Clopper–Pearson method.

**Table 2:** Clinical outcomes and complications.

Variable	ACAO (*N* = 67)
Total 90-day mortality all-cause, no. (%; 95% CI)	19 (28.4%; 18.1–40.6)
Mortality during hospitalization, no. (%; 95% CI)	12 (17.9%; 9.6–29.2)
Mortality 90 days after hospital discharge, no. (%; 95% CI)	7 (10.5%; 4.3–20.3)
Favorable outcome at 90 days (mRS 0–2) (%; 95% CI)	29 (43.3%; 31.3–55.9)
Any procedural complications * (%; 95% CI)	11 (16.4%; 8.5–27.5)
Intracranial hemorrhage, no. (%; 95% CI)	25 (37.3%; 25.8–49.9)
HI 1, no. (%)	10 (14.9)
HI 2, no. (%)	6 (9.0)
PH 1, no. (%)	2 (3.0)
PH 2, no. (%)	3 (4.5)
SAB, no. (%)	4 (6.0)
sICH, no. (%)	6 (9.0)
Stent occlusion at hospital discharge, no. (%)	2/58 (3.4)
IV eptifibatide	1
No eptifibatide	1
Stent occlusion at 90 days, no. (%)	4 (6.0)
IV eptifibatide	4
No eptifibatide	0

* Perforation (*N* = 4), internal carotid artery dissection (*N* = 1), Embolic new territory (*N* = 6). ACAO, atherosclerotic carotid artery occlusion; mRS, modified Rankin Scale score; HI, hemorrhagic infarction; PH, parenchymal hematoma; SAB, subarachnoid hemorrhage; sICH, symptomatic intracranial hemorrhage. Data are presented as *n* (%). Proportions include exact 95% confidence intervals calculated using the Clopper–Pearson method.

**Table 3:** Comparing baseline characteristics and outcome of patients treated intravenously with eptifibatide vs. without.

Outcome	*N* = 67	IV Eptifibatide (*n* = 35)	No Eptifibatide (*n* = 32)	*p* Value
Age mean (±SD)	74 (±15)	72 (±10.9)	75 (±9.7)	0.221
NIHSS on admission, median (IQR)	11.5 (7–16)	12 (7–12)	11 (7–15)	0.399
ASPECTS, median (IQR)	8 (7–9)	8 (6.75–9)	8 (7–9)	0.61
Onset to puncture min, median (IQR)	255 (165–691)	257 (150–663)	221 (159–570)	0.333
Procedure time min, median (IQR)	75 (54–114)	60 (55–115)	74 (60–113)	0.781
IV thrombolysis rtPA, no. (%; 95% CI)	26 (38.8%; 27.1–51.5)	14 (40.0%; 23.9–57.9)	12 (37.5%; 21.1–56.3)	0.834
sICH no. (%; 95% CI)	6 (9.0%; 3.4–18.5)	6 (17.1%; 6.6–33.6)	0 (0.0%; 0.0–10.9)	0.021
Parenchymal hematoma type 1–2, no. (%)	5 (7.5%; 2.5–16.6))	4 (11.4%; 3.2–26.7)	1 (3.1%; 0.1–16.2)	0.196
90-day mortality *, no. (%; 95% CI)	19 (28.4%; 18.1–40.6)	10 (28.6%; 14.6–46.3)	9 (28.1%; 13.7–46.7)	0.968
90-day favorable outcome *, no. (%; 95% CI)	29 (43.3%; 31.3–55.9)	17 (48.6%; 31.9–65.6)	12 (37.5%; 21.1–56.3)	0.361
mTICI 2b-3′, no. (%; 95% CI)	37/65 (56.9%; 44.1–69.1)	23 (65.7%; 47.8–80.9)	14 (43.8%; 26.4–62.3)	0.071
mTICI 3′, no. (%; 95% CI)	27/65 (41.5%; 29.4–54.4)	11 (31.4%; 16.9–49.3)	16 (50.0%; 31.9–68.1)	0.122

* Three patients were lost to follow-up; no mTICI score was documented for two patients. NIHSS, National Institutes of Health Stroke Scale; ASPECTS, Alberta Stroke Program Early CT Score; rtPA, recombinant tissue Plasminogen Activator; sICH, symptomatic intracranial hemorrhage; mTICI, modified Thrombolysis in Cerebral Infarction. Data are presented as mean ± SD, median (IQR), or *n* (%). Proportions include exact 95% confidence intervals calculated using the Clopper–Pearson method. Comparisons were made using Fisher’s exact test and the Mann–Whitney-U test.

**Table 4:** Logistic regression analyses for symptomatic intracranial hemorrhage (sICH).

Model	Predictor	Odds Ratio	95% CI	*p*-Value
Univariable	Eptifibatide	1.9	0.3–11.4	0.465
Age-adjusted (two-parameter model)	Eptifibatide	2.2	0.4–13.1	0.395
	Age (per year)	1.1	1.0–1.1	0.301
NIHSS-adjusted (two-parameter model)	Eptifibatide	2.1	0.4–12.2	0.429
	NIHSS on admission (per point)	0.9	0.8–1.1	0.389

Because only six patients experienced sICH, we restricted logistic regression to univariable and parsimonious two-parameter models (exposure plus one covariate) to avoid overfitting. OR indicates odds ratio; CI, confidence interval; NIHSS, National Institutes of Health Stroke Scale. All models used sICH as the dependent variable and periprocedural eptifibatide administration as the primary independent variable.

## Data Availability

The data supporting this study’s findings are available on request from the corresponding authors. The data are not publicly available due to privacy or ethical restrictions.

## Supplementary Materials

The following supporting information can be downloaded at https://www.mdpi.com/article/10.3390/diagnostics16091281/s1, Table S1: Characteristics of patients with symptomatic intracranial hemorrhage (sICH).

## Abbreviations

The following abbreviations are used in this manuscript:

ACAD	Atherosclerotic carotid artery disease
CAS	Carotid artery stenting
ETIS	Endovascular Treatment in Ischemic Stroke
ICA	Internal carotid artery
IVT	Intravenous thrombolysis
LVO	Large vessel occlusion
MT	Mechanical thrombectomy
ICH	Intracranial hemorrhage
TITAN	Thrombectomy in Tandem lesions
TO	Tandem occlusions
