# Supplementary material for: Adaptive Antithrombotic Strategy for Tandem Occlusion Stroke: Escalating Therapy After Thrombectomy and Stenting
Source: Diagnostics (Basel). 2026 Apr 24;16(9):1281. doi: 10.3390/diagnostics16091281 (PMC13162882; doi:10.3390/diagnostics16091281)
Supplement: Supplementary file 1 [file diagnostics-16-01281-s001.zip › diagnostics-4167411-supplementary.pdf]

diagnostics

MDPI

**Supplementary Table S1.** Characteristics of patients with symptomatic intracranial hemorrhage (sICH)							
Patient	Age (years)	Sex	IVT with rtPA	Heparin given	Eptifibatide (h)	Aspirin loading	ASPECTS
1	74	M	X	X	3	X	7
2	69	F		X	26	X	7
3	85	F	X		2.5	X	8
4	80	M	X	X	1	X	10
5	82	M	X	X	5.5	X	9
6	76	F		X	1	X	6

ASPECTS, Alberta Stroke Program Early CT Score; IVT, intravenous thrombolysis; rtPA, recombinant tissue Plasminogen Activator.
